# The effect of reward on motor learning: different stage, different effect

**DOI:** 10.3389/fnhum.2024.1381935

**Published:** 2024-03-12

**Authors:** Jingwang Zhao, Guanghu Zhang, Dongsheng Xu

**Affiliations:** ^1^School of Rehabilitation Science, Shanghai University of Traditional Chinese Medicine, Shanghai, China; ^2^Institute of Rehabilitation Medicine, Shanghai University of Traditional Chinese Medicine, Shanghai, China; ^3^Engineering Research Center of Traditional Chinese Medicine Intelligent Rehabilitation, Ministry of Education, Shanghai, China; ^4^Department of Rehabilitation Medicine, Shuguang Hospital, Shanghai, China

**Keywords:** motor learning, reward, rehabilitation, memory retention, motivation, neural circuit, stages

## Abstract

Motor learning is a prominent and extensively studied subject in rehabilitation following various types of neurological disorders. Motor repair and rehabilitation often extend over months and years post-injury with a slow pace of recovery, particularly affecting the fine movements of the distal extremities. This extended period can diminish the motivation and persistence of patients, a facet that has historically been overlooked in motor learning until recent years. Reward, including monetary compensation, social praise, video gaming, music, and virtual reality, is currently garnering heightened attention for its potential to enhance motor motivation and improve function. Numerous studies have examined the effects and attempted to explore potential mechanisms in various motor paradigms, yet they have yielded inconsistent or even contradictory results and conclusions. A comprehensive review is necessary to summarize studies on the effects of rewards on motor learning and to deduce a central pattern from these existing studies. Therefore, in this review, we initially outline a framework of motor learning considering two major types, two major components, and three stages. Subsequently, we summarize the effects of rewards on different stages of motor learning within the mentioned framework and analyze the underlying mechanisms at the level of behavior or neural circuit. Reward accelerates learning speed and enhances the extent of learning during the acquisition and consolidation stages, possibly by regulating the balance between the direct and indirect pathways (activating more D1-MSN than D2-MSN) of the ventral striatum and by increasing motor dynamics and kinematics. However, the effect varies depending on several experimental conditions. During the retention stage, there is a consensus that reward enhances both short-term and long-term memory retention in both types of motor learning, attributed to the LTP learning mechanism mediated by the VTA-M1 dopaminergic projection. Reward is a promising enhancer to bolster waning confidence and motivation, thereby increasing the efficiency of motor learning and rehabilitation. Further exploration of the circuit and functional connections between reward and the motor loop may provide a novel target for neural modulation to promote motor behavior.

## 1 Introduction

The restoration of motor function is a paramount objective in neural rehabilitation. Rehabilitation involving movement is marked by prolonged exercises and gradual recovery, particularly for the fine motor skills of the distal extremities. Numerous studies have concentrated on various aspects of motor training, including motor paradigms, training duration, intensity, and types of exercises (active, passive, or resistive), aiming to enhance motor function (Breceda and Dromerick, 2013; Kim et al., 2020; Vidaurre et al., 2023). However, motivation is significantly neglected in both clinical practice and research studies on motor rehabilitation (Robertson, 2013; Verrienti et al., 2023). A common issue is that patients often experience frustration and lack enthusiasm to attend training sessions due to post-stroke depression or the prolonged training period with minimal progress (Qian et al., 2019). Low motivation leads to decreased efficiency and unsatisfactory motor improvement. As is widely recognized, rewards are potent motivators. Consequently, researchers have heightened their scientific interest in strategically employing rewards to facilitate motor rehabilitation. Diverse rewards, including monetary compensation, verbal praise, virtual reality and music, are known to affect motivation and influence performance in a variety of motor tasks (Widmer et al., 2016; Quattrocchi et al., 2017; Shiomi et al., 2020; Song et al., 2020; Vassiliadis et al., 2021, 2022; Sporn et al., 2022; Yin et al., 2023a,b). Studies support that rewards can promote motor learning and prolong memory retention in both healthy individuals and stroke patients. However, some studies have suggested inconsistent or even opposite results (Johannsen, 1962; Galea et al., 2015; Yin et al., 2023a).

Motor learning is a comprehensive term that encompasses various phenomena inherent in the learning process of motor behavior. The breadth of the motor learning concept, the diversity of motor paradigms employed in learning studies, and the inconsistency of findings have left researchers perplexed regarding the precise role of reward in motor learning, not to mention the potential mechanisms and future research directions. While the precise behavioral repertoires needed for distinct motor paradigms vary, common features like learning stage and component imply the need for shared conceptual substrates across a broad spectrum of motor tasks. Therefore, in this review, we initially provide a brief overview of the connotations of motor learning from the perspectives of type, component, and stage. Subsequently, we summarize the impact of rewards on various stages of motor learning and explore the underlying mechanisms at the levels of behavior and microcircuit.

## 2 The types, components and stages of motor learning

In real contexts, it may takes thousands of hours or more to grasp a complex motor skill and become an expert, such as a top football player. However, in research and experimental settings, motor paradigms primarily involve simple learning tasks that can be rigorously investigated under experimental conditions. These studies highlight more elementary forms of learning, providing foundational insight into the nature of complex, real-world skill learning.

There are two long-standing types of motor learning that have been considered by research - motor sequence and motor adaptation. Sequential motor learning is defined as choosing a goal in a task context, planning and selecting the correct action based on external and internal information, and executing the action accurately and precisely (Krakauer et al., 2019). It typically involves the generation of a new movement pattern and is characterized by shifts in the speed–accuracy relationship. Both simple sequences such as finger tapping and complex sequences (commonly called skills), such as throwing a basketball and playing piano are considered. The most commonly used sequential motor paradigm is the serial reaction time task, which requires arm reaching (Moisello et al., 2009), finger pressing (Tzvi et al., 2014) and foot stepping (Du and Clark, 2018) toward targets in the instructed order. Substantial training is required to learn a motor skill successfully, but the acquired motor memory is retained for a long time once the skill is grasped.

Motor adaptation entails maintaining performance in response to an ever-changing environment or changes to the body itself by adjusting an already well-learned action, while the goal of the action remains the same (Krakauer et al., 2019). Common motor adaptation paradigms include the force-field adaptation task (force field adaptation) (Shadmehr and Mussa-Ivaldi, 1994), visuomotor rotation (VMR) tasks (visuomotor adaptation) (Mazzoni and Krakauer, 2006), saccadic tasks (vestibulo-ocular reflex adaptation) (Pham et al., 2022), split-belt treadmill training (gait adaptation) (Stubbs and Gervasio, 2012) and speech production adaptation tasks (Parrell et al., 2017).

Explicit and implicit learning are the fundamental components of motor learning. There are two information processing pathways in motor learning: the spatial processing stream and motor processing stream. The former encodes the visuospatial coordinates of the movement, usually under explicit attention and cognition, through cerebellar-cortical and parietal–frontal cortex, preferentially dominated in the early stages; the latter encodes the motor plan that is enacted by the muscles implicitly through cerebello- and striato-motor-cortex loops (Doyon et al., 2003).

**Figure 1:**
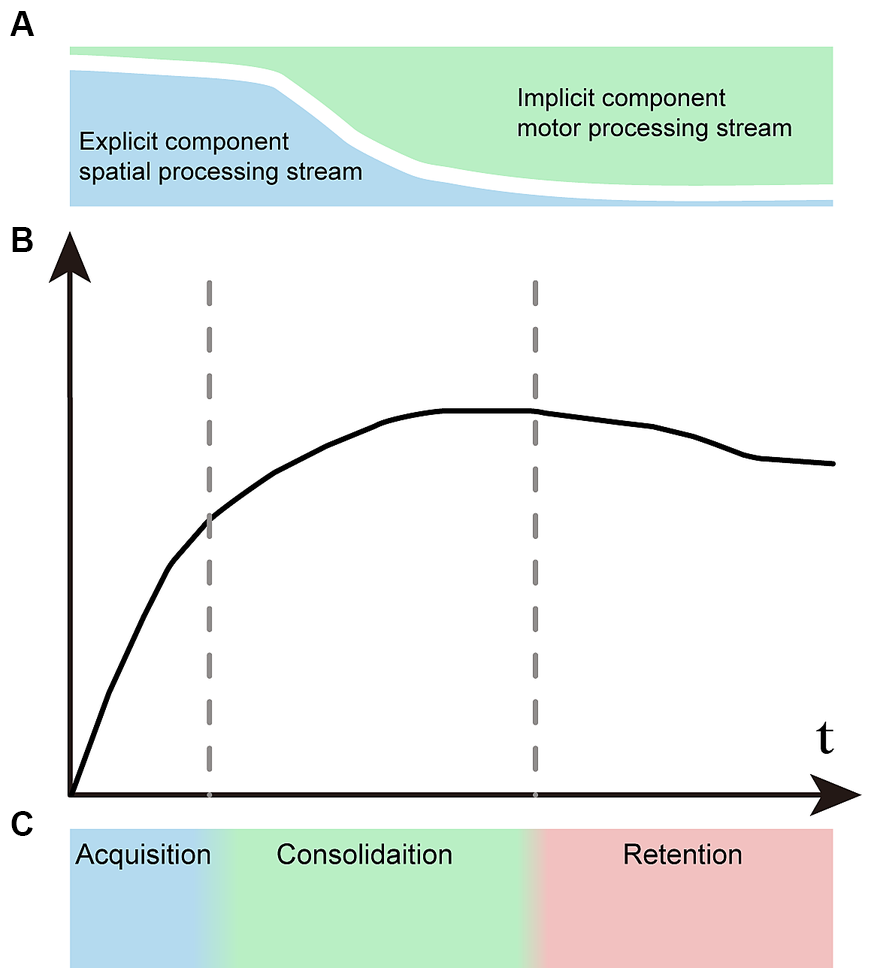
Representation of the concept of motor learning. **(A)** Explicit and implicit components vary proportionally at different stages of motor learning. Explicit learning predominates in the early phase and significantly diminishes in the later phase. Conversely, implicit learning follows the opposite pattern. **(B)** The learning curve of motor sequences undergoes changes across various stages. **(C)** There is no distinct boundary between the acquisition, consolidation, and retention stages of sequence learning.

Explicit motor learning involves cognitive participation and is based on working memory throughout the learning process (Seidler et al., 2012). Implicit motor learning takes place without awareness and in the absence of verbal knowledge of the performed motor task. In addition, some processes take place implicitly when the associated movements are nearly automatic. The final state of motor learning may be implicit skills, but explicit cognitive function contributes to almost all stages of motor learning, particularly in the initial learning period (Therrien and Wong, 2021). Typically, explicit learning precedes implicit learning, but the majority of the learning process involves both explicit and implicit information processing in parallel in varying proportions (Figure 1A).

Owing to the different signatures of explicit and implicit learning components, the motor learning process has time-dependent characteristics. The explicit visual–spatial component is learned quickly in the initial stage, and the implicit processing component increases gradually in the later stage. Correspondingly, we can see two distinct time constants in the learning curve: the early learning stage showing rapid improvement, followed by slower improvements during the later stage (Hikosaka et al., 1999; Luft and Buitrago, 2005; Seidler et al., 2012; Dahms et al., 2020) (Figure 1B). Motor learning is artificially divided into three primary stages: the acquisition stage, the consolidation stage and the retention stage (Figure 1C). There are no clear boundaries between different stages, and the two information processing streams associated with learning are active in parallel in varying proportions that depend on task demands. Although the learning processes are intertwined, explicit processes typically acquire fast and early, while implicit processes mostly drive slower learning at the later stage.

Motor learning is driven by different types of errors: sensory prediction errors, task errors and reward prediction errors (Izawa and Shadmehr, 2011; Palidis et al., 2019; Codol et al., 2020). Sensory prediction error is the error observed or captured by our sensory organs, i.e., the mismatch between the movement we “see” and the actual movement we perform (e.g., the mismatch between cursor and hand position in the VMR task). Implicit learning mechanisms maintain the level of motor performance under fluctuating conditions using sensory prediction error and depend on the cerebellum. Task error is defined as the discrepancy between the actual movement outcome and the motor target, which acts as a vectorial signal to drive motor learning, providing information about the error direction and magnitude. Task errors that are only reported as the binary success/failure of the movement are called reward prediction errors. The participant always presumably predicts that their movement is successful in motor tasks (the rewarding result). Using a Visuomotor Rotation (VMR) task as an illustration, individuals guided a cursor to visual targets by moving it with one hand, either through a robotic device or a motion tracking system, executing rapid aiming motions. The cursor’s path deviated from the actual hand movement, pivoting around the initiation point, thereby introducing an unfamiliar visuomotor shift and an error in performance. To adapt to this new setting and regain precise control, participants were required to adjust their hand’s movement trajectory (Galea et al., 2015). In this motor activity, the sensory prediction error refers to the discrepancy between the actual hand position and the cursor’s location, whereas the task error denotes the divergence between the target’s location and the hand’s position. The outcomes of the aiming attempts are classified based on the magnitude of the task error and are rewarded accordingly (either with money or tokens).

## 3 Effect of reward on different stages of motor learning

Avoiding punishment and pursuing reward are strong motivations that affect human behavior. Rewarding stimuli induce pleasure and generate approaching behaviors, eventually leading to behavioral reinforcement. The expectation of obtaining a reward or avoiding punishment motivates learning and decision making (Rigoux and Guigon, 2012; De Comite et al., 2022). Many studies focus on the rewards used to reinforce motor learning and have yield inconsistent, or even contradictory, conclusions. The diverse motor paradigms, experimental subjects, reward content and experimental time of these studies alongside many other factors make it challenging to clearly understand the effect of reward on motor learning within a cohesive framework. Given these situations and based on the above analysis of time-dependent characteristics in motor learning, we propose to summarize the effect of rewards on the three stages of motor learning.

From the view of the learning curve, we assess the effect on motor learning through three important indicators: learning rate, learning extent and decay rate. The learning rate reflects how quickly participants learn a skill or adapt to a new perturbation during early learning. Taking adaptation as an example, according to the single-rate-state-space model defined as [*x*(*k*) = *Ax*(*k*-1)-*Be*(*k*-1)], the learning rate is the fraction of the error that is corrected from one trial to the next (parameter B); this parameters represents the steep slope of the learning curve for the acquisition stage. The learning extent indicates how well participants learn the task during consolidation and reflects the slower slope and the plateau of the learning curve. The degree of memory decay on each trial (parameter A) estimates retention (Thoroughman and Shadmehr, 2000; Donchin et al., 2003; Cheng and Sabes, 2006).

### 3.1 Rewards accelerate learning speed (acquisition stage)

The acquisition stage is characterized by rapid performance improvements, during which the sequence of movement is learned quickly; learning in this state is mediated by improved encoding of the spatial sequential component. The learning process demands working memory and attentional resources (Hikosaka et al., 2002).

Earlier studies demonstrated that reward alone did not enhance the learning rate in the VMR task, whereas punishment alone, whether graded or binary, accelerated motor learning (Galea et al., 2015). Moreover, reward combined with punishment not only accelerated the learning rate but also increased the learning extent. Nikooyan et al. suggested that reward feedback alone can drive motor adaptation (without increasing the rate beyond that of the control group), and the combination of reward and sensory feedback accelerates learning (Nikooyan and Ahmed, 2015).

**Figure 2:**
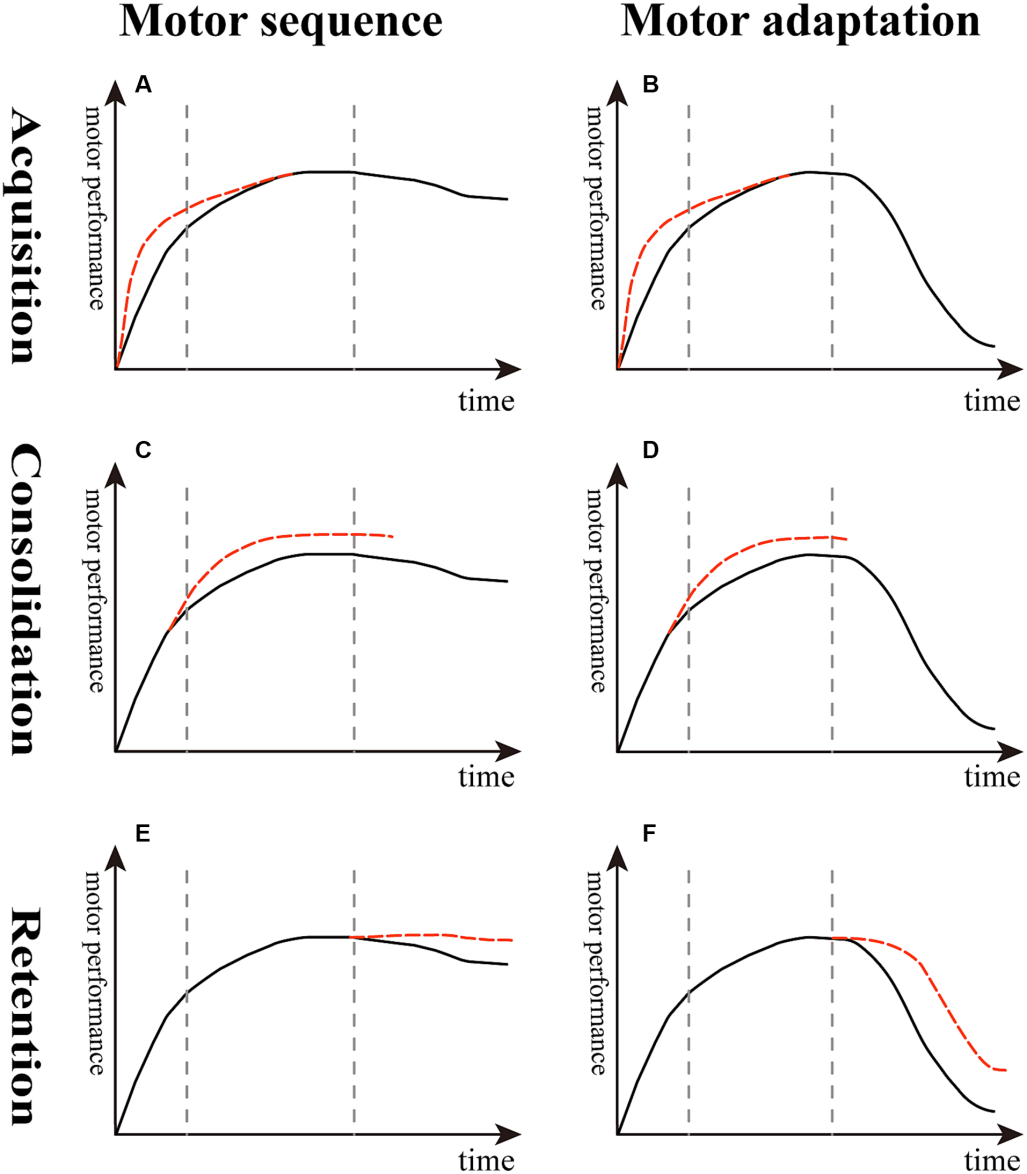
Effect of reward on different stages of sequence learning and motor adaptation. **(A,B)** The slope of the red dotted line is higher than the black solid line, indicating that reward boosts the learning rate in the acquisition stage. **(C,D)** The top of the red dotted line is higher than the black solid line, suggesting that reward enhances learning extent in the consolidation stage. **(E,F)** The decline of the red dotted line is lesser than the black solid line, meaning that reward decreases memory decay and improves memory retention in the retention stage of motor sequence and adaptation.

In contrast, other studies suggested that reward alone boosts or accelerates learning speed in sequence learning paradigms (Figure 2A) and motor adaptation tasks (Figure 2B). Anderson et al. showed that participants with monetary incentives have a higher learning rate in a discrete motor sequence task because the reward enhances motivation (Anderson et al., 2020). Additionally, Sebastian Sporn et al. dissociated the effects of different types of rewards, namely, performance feedback and monetary incentives, through a novel motor task, and the results demonstrated that monetary incentives alone rapidly shortened movement time, whereas feedback after correct responses primarily improved learning-related movement time performance. Importantly, pairing both monetary incentives and feedback after correct responses enhanced movement time performance and improved fusion of movements. The fusion of movements enhanced both motor speed and efficiency by increasing movement smoothness. These reward-based improvements lasted for 24 h after canceling the reward (Sporn et al., 2022).

In conclusion, a consensus remains elusive regarding whether reward can expedite motor learning. The inconsistent outcomes of prior studies could stem from several factors. Primarily, the motor paradigms employed in most studies are comparatively straightforward, allowing participants to quickly reach a plateau, thereby blurring distinctions between the reward and neutral/control groups. Secondly, potentially for the sake of experimental convenience, many studies enlist healthy young participants, and the limited age range represented may exhibit a specific sensitivity or preference for reward or punishment. Thirdly, though not conclusively, almost all forms of reward are monetary, typically comprising a fixed amount and occasionally a performance-based bonus. These rewards may not consistently kindle sufficient motivation, or studies may have overlooked the heterogeneity among different participants in terms of their responses to rewarding stimuli, thereby introducing significant variation. Consequently, further studies are imperative to precisely elucidate the exact role of reward in motor learning.

### 3.2 Rewards enhance learning extent (consolidation stage)

In the consolidation stage, the learning rate decelerates, and acquired information stabilizes (Hikosaka et al., 1999). Performance gradually enhances through practice, gaining resistance to interference. The brain assimilates visual–spatial and sensorimotor information, utilizing external cues to execute actions. The shift from the “acquisition” stage to the “consolidation” stage is seamless, lacking a distinct boundary.

In an arc-pointing sequence task, monetary reward following good performance resulted in better consolidation than performance feedback alone (Widmer et al., 2016) (Figure 2C). In a VMR-based reaching task using a robotic arm, compared with the neutral group, the reward group of chronic stroke patients showed greater adaptation and readaptation (Figure 2D) and adapted to a similar degree as healthy controls (Quattrocchi et al., 2017). In another study using a VMR center-out reaching task performed in healthy young adults, the results showed that reward combined with punishment, rather than reward alone, increased the extent of learning (Yin et al., 2023b).

The potential age-related bias may be a contributory cause for the inconsistent results above since young adults are more sensitive to errors (i.e., more aversive to punishment), thus highlighting the effect of punishment, rather than reward, on motor adaptation; nevertheless, patients with chronic stroke (older adults) demonstrated more sensitivity to reward (Marschner et al., 2005; Quattrocchi et al., 2017). Another key factor that directly affects motor learning is the timing at which reward is delivered following movement execution. Short reward delays induced continuous improvements in performance and greater overnight consolidation. However, for a longer reward delays, the learning rates were initially high, but then learning reached a plateau more quickly and performance was lower at the end of training. Overnight memory consolidation is also reduced with longer reward delays (Vassiliadis et al., 2022).

### 3.3 Rewards extend memory retention (retention stage)

During the retention stage, the movement is performed with decreasing attention until it is almost entirely implicit and becomes “automatic”; the motor learning is optimized at a slow rate. Sensorimotor information and visuospatial information are integrated together efficiently, and the primary motor cortex (M1) creates “motor maps” to control muscles collaboratively to optimize movements (Hirano et al., 2015). The motor information becomes robust and stable as the motor map optimization is accomplished. Motor skills are learned slowly over months or even years but, once mastered, are retained for long periods with minimal decay. In motor adaptation paradigms, there are aftereffects when the perturbation is removed, but these aftereffect appear to be inherently transient and rapidly decay to baseline in subsequent trials (Luft and Buitrago, 2005). The different retention of sequence learning and adaptation may be due to the respective mechanism of memory storage.

Compared with the inconsistent results reflecting the acquisition and consolidation stages described above, there seems to be a consensus that reward improves short-term and long-term memory retention both in healthy young and stroke patients. First, Wächter et al. found that punishment enhanced motor performance, whereas reward led to greater memory retention in a sequential motor paradigm (Wachter et al., 2009) (Figure 2E). Abe et al. examined the effect of monetary reward on a tracking isometric pinch force task, demonstrating that the group with training with rewards showed greater improvements both overnight and 30 days later compared with the punishment and neutral groups; they suggested that this enhanced long-term retention was driven by offline memory gains (Abe et al., 2011). Later studies supported the idea that participants undergoing training with reward feedback exhibit less memory decay by demonstrating the increased short-term memory retention in a VMR task both in healthy young adults and chronic stroke older patients (Galea et al., 2015; Quattrocchi et al., 2017) (Figure 2F).

## 4 The underlying mechanisms

### 4.1 From the level of behavior

Generally, rewards and incentives increase motivation, which drives individuals to expend more energy to execute movements faster and more accurately, thus optimizing the speed–accuracy trade-off function (Takikawa et al., 2002; Manohar et al., 2015; Summerside et al., 2018). Specifically, rewards boost motor execution by improving motor dynamics and kinematics. In terms of dynamics, studies have revealed that reward could increase velocity, shorten movement times and speed up the movement tempo in sequence learning (Wachter et al., 2009). Regarding motor kinematics, reward feedback minimizes movement time by fusing neighboring sequential movements together more efficiently through increased smoothness (Sporn et al., 2022). In addition, reward feedback could enhance limb stability by increasing limb stiffness to sustain motor accuracy (Codol et al., 2020).

The presentation of momentary reward is associated with changes in M1 activity (Thabit et al., 2011). In a study utilizing a slot machine to simulate variable monetary rewards without involving any movement, the heightened expectation preceding the eventual reward delivery correlated with increased intracortical inhibition in M1; a substantial reward intensified this effect (Kapogiannis et al., 2008). In motor tasks, there is typically a transient suppression of corticospinal excitability during preparation, and the anticipation of reward induces a linear decrease in motor-evoked potential amplitude and a larger decrease in corticospinal excitability, suggesting a similar inhibition in M1 (Bundt et al., 2016). EEG evidence supports that a higher reward probability induces a greater lateralized readiness potential during motor preparation, indicating a greater effort in executing the correct movement (Chen et al., 2022). These transitory inhibition effects appear to represent the storage of a more robust motor plan to prepare for the movement and generate a vigorous and improved motor execution.

In our daily lives, environments abound with uncertainty, prompting continuous adaptations in our behaviors based on the evolving relationship between actions and rewards. Recently, Tecilla et al. demonstrated that motor vigor improves in the context of varying reward probability, employing a validated hierarchical Bayesian model of a sequential motor task. Stronger prediction trends of reward contingency resulted in a faster performance tempo on a trial-by-trial basis without significant alterations in reaction times. Importantly, these results held true for both healthy young and elderly adults, as well as individuals with Parkinson’s disease (Tecilla et al., 2023).

In a word, reward serves to facilitate movement recruitment in advance, enhancing motor preparation. Furthermore, the anticipation of reward elevates motor vigor, expediting all actions during motor execution. Ultimately, this leads to a swifter performance of the action and smoother movement coordination, thereby improving motor efficiency, accelerating the learning rate, and enhancing the overall extent of learning.

### 4.2 From the level of neural circuit

It is challenging to formulate the mechanistic framework if we analyze the phenomenon solely from the perspective of either the motor circuit or the reward circuit. Therefore, we suggest that identifying the mutual and shared brain structures between the two circuits may be the key to unraveling this mystery. First, we briefly introduce classical concepts and recent research on the reward circuit. Then, we analyze the potential “interface” between the reward and motor circuits, along with the evidence supporting it.

The reward circuit, a complex network comprising cortical and subcortical regions, plays a pivotal role in incentive learning, adaptive behaviors, and decision-making. The ventral tegmental area (VTA) and the nucleus accumbens (NAc) emerge as cornerstones of this circuit.

The VTA is a heterogeneous brain region located in the ventral part of the midbrain, which is widely accepted as the starting point from where the reward circuit begins (Salamone, 1994). It consists of three kinds of neurons: dopamine (DA) neurons (60–65%), γ-amino butyric acid (GABA) neurons (30–35%), and a small group of glutamatergic neurons (2–3%) (Yamaguchi et al., 2007; Dobi et al., 2010). The VTA DA neurons project preferentially to NAc (ventral striatum) (Voorn et al., 2004; Shen et al., 2008). Studies have proved that these dopamine signals encode reward prediction error—the disparity between actually obtained and expected reward (D'Ardenne et al., 2008; Flagel et al., 2011). The dopaminergic prediction error serves as a teaching signal that modifies glutamatergic inputs and dopaminergic inputs in the striatum during unexpected rewards (Reynolds and Wickens, 2002; Hyman et al., 2006). Therefore, actions that bring an unexpected reward tend to be pursued, leading to positive reinforcement.

The NAc, positioned as the principal component of the ventral striatum, serves as a primary downstream target of the VTA dopamine projection. Comprising GABAergic medium spiny neurons (MSNs) expressing D1-like or D2-like receptors, the NAc exhibits distinct direct and indirect pathways. The direct pathway involves projections from D1 MSNs to the VTA, while both types of MSNs send projections to the ventral pallidum (VP) first and then to the VTA in the indirect pathway (Gerfen et al., 1990). NAc functions as an interface between the limbic and motor systems, receiving upstream information from the limbic system and projecting to the ventral pallidum and other motor effector areas to translate the signals into actions (Floresco, 2015). VTA dopaminergic signals may act as a modulator between upstream glutamatergic inputs (from the prefrontal cortex, hippocampus, amygdala, and thalamus) and the NAc MSN neurons, integrating upstream synaptic information and encoding them as reward prediction error or motivation value in the NAc.

Several other brain regions are involved in reward processing, apart from the VTA and the NAc, such as the medial prefrontal cortex (mPFC), thalamus, ventral hippocampus, basolateral amygdala, and the newly uncovered cerebellum (Malvaez et al., 2019; Kostadinov and Hausser, 2022). mPFC is associated with executive control and the modulation of behaviors such as planning and seeking to pursue rewarding stimuli (Ma et al., 2014). Beier et al. discovered several previously unidentified pathways from the mPFC to VTA-DA and to the lateral NAc, demonstrating direct top-down executive control (Beier et al., 2015). Both the ventral hippocampus and basolateral amygdala project glutamatergic synapses to the NAc. The ventral hippocampus mainly involves in processing emotional information to influence goal-directed behavior (Charara and Grace, 2003; Sherafat et al., 2020). Activating projections from the basolateral amygdala to the NAc contributes to reward seeking and facilitates positive reinforcement (Stuber et al., 2011). The paraventricular nucleus (PVT) is the midline thalamic nucleus of the thalamus, which is also proven to have glutamatergic projections into the NAc (Zhu et al., 2016), and direct activation of the PVT–NAc pathway induces aversive behavior (Browning et al., 2014).

The cerebellum, traditionally regarded as a dedicated motor structure, has undergone a paradigm shift in recent research, revealing its involvement in non-motor functions. Although historically overlooked in the context of reward, contemporary studies have unveiled critical non-motor functions of the cerebellum. Notably, Carta et al. identified monosynaptic excitatory projections from the cerebellar nuclei to the VTA, encompassing both dopaminergic and non-dopaminergic pathways. These projections exert a potent influence on the reward circuit, thereby impacting social behavior. Optogenetic stimulation of the cerebellar-to-VTA projection resulted in a robust increase in VTA neuron activity. This activation proved sufficient to induce both short-term and long-term place preferences, providing compelling evidence for the rewarding nature of this cerebellar pathway (Carta et al., 2019). Consistent with these findings, additional studies further supported the rewarding properties of the cerebellum (Larry et al., 2019; Lixenberg et al., 2020).

### 4.3 The interface between reward and motor circuit

Building upon the previously discussed perspective and the contextualization of the reward circuit, two pivotal intersections with the motor circuit come to the forefront: the NAc and VTA. The NAc, being a core region in the reward circuit, remains intricately involved in the cortico-striatum-thalamo-cortical loop, serving as a central component of the ventral striatum. On the other hand, the VTA sends direct dopaminergic projections to the M1, a region crucial for acquiring new skills and executing movement sequences (Luft et al., 2004). Our intent is to elucidate the underlying mechanisms from these dual perspectives.

**Figure 3:**
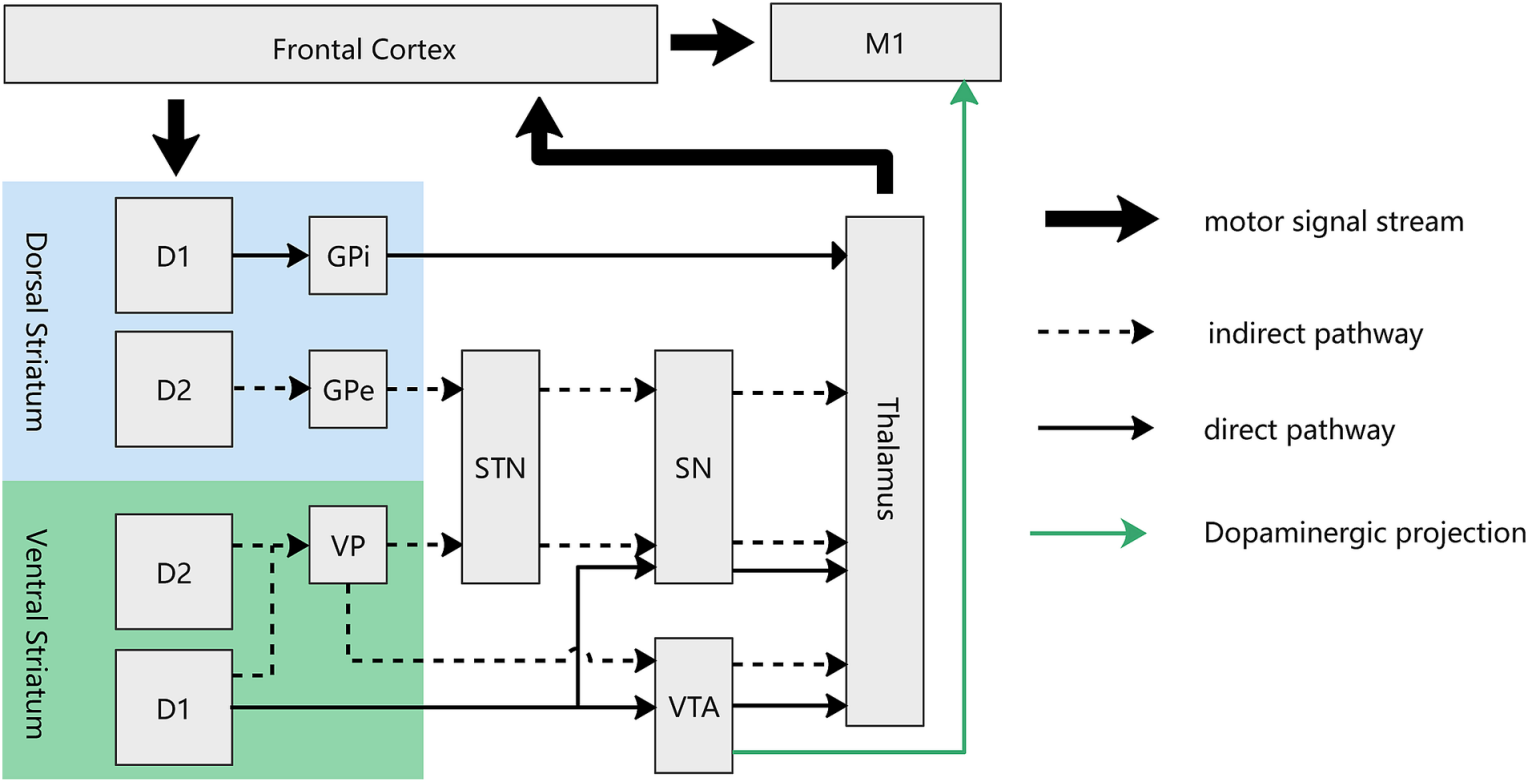
Circuit diagram of direct and indirect pathway of both dorsal and ventral striatum in the motor loop. For the ventral striatum (NAc), the direct pathway, mediated by D1-MSNs, involves projections from the NAc to the VTA and SN, which subsequently project to the thalamus. The indirect pathway, mediated by both D1- and D2-MSNs, traverses the ventral pallidum before reaching the VTA and STN. There exist dopaminergic projections from the VTA to M1, mediating the reward-based motor learning. D1, D1-MSN, D2: D2-MSN; GPe, external globus pallidum; GPi, interal globus pallidum; VP, ventral pallidum; STN, Subthalamus nucleus; SN, Substantia nigra; VTA, ventral tegmental area.

In the ventral striatum, a direct/indirect pathway akin to that in the dorsal striatum extends from the NAc to the thalamus. The direct pathway entails projections from the NAc to the VTA (Xia et al., 2011; Bocklisch et al., 2013), which subsequently projects to the thalamus. In contrast, the indirect pathway traverses the ventral pallidus before reaching the aforementioned regions. Much like the dorsal striatum, the direct pathway of the ventral striatum is exclusively mediated by D1-MSNs, while the indirect pathway involves both D1- and D2-MSNs (Soares-Cunha et al., 2020) (Figure 3). The GABAergic (inhibitory) projections from the NAc to the VTA form part of a feedback loop that regulates dopaminergic activity in the VTA. When activated, neurons in the NAc can inhibit VTA dopamine neurons, thereby modulating the release of dopamine in the NAc itself and other target areas, such as the thalamus. The standard “rate” model provides a foundational understanding that the thalamus functions as a “leash” to restrain cortical activity. While stimulation and lesions of thalamic regions lead to similar outcomes (Marsden and Obeso, 1994), suggesting that the thalamus may facilitates a balance between excitation and inhibition in cortical areas and actively participates in the modulation of cortical processing and the integration of motor and cognitive functions. Future research are needed to elucidate these intricate relationships further to unravel the precise mechanisms by which these neural circuits contribute to reward processing and motor learning. Our deduction posits that rewarding stimuli activate specific NAc MSNs, particularly favoring D1-MSNs over D2-MSNs. This activation is instrumental in modulating the VTA and thalamus (alongside other downstream regions), ultimately fine-tuning the equilibrium between excitatory and inhibitory signals within cortical regions. Furthermore, it facilitates the integration of reward and motor signals, underscoring a sophisticated neural mechanism underlying reward processing and motor function coordination. This regulatory mechanism effectively amplifies motivational value and augmenting motor behavior. Traditionally, the prevailing belief was that the NAc primarily governs motivation-driven effort without direct involvement in motor control. However, recent findings by Sawada et al. in non-human primates challenge this notion. The research team recorded brain activity from the NAc to the sensorimotor cortex during the recovery of finger movements after spinal cord injury in four macaque monkeys. Early in the recovery period, NAc inactivation resulted in reduced Gamma oscillation in the sensorimotor cortex, leading to a transient decline in finger dexterity (Sawada et al., 2015). This compelling evidence lends support to our hypothesis that the NAc serves as the interface between the motor and reward circuits, mediating the impact of reward on motor learning. Although the exact neural circuit through which the NAc exerts its effects remains undetermined, this study significantly enhances our understanding of the NAc’s direct involvement in controlling finger movements.

In another dimension, the pivotal role of dopaminergic signals originating from the VTA in enhancing motor skill learning is evident through their influence on synaptic plasticity in the M1. Multiple studies substantiate the existence of dopaminergic connections from the VTA to the M1. Retro-tracing from the M1 has identified dopaminergic neurons in the VTA, and electrical stimulation of the VTA induces c-fos expression in the M1 cortex. Additionally, the outcomes of VTA electrical stimulation can be effectively blocked by D1 and D2 antagonists. Notably, investigations into VTA dysfunction have revealed no discernible impact on previously acquired motor skills (Hosp et al., 2011; Hosp and Luft, 2013). These collective findings support the notion that dopaminergic projections originating from the VTA initiate long-term potentiation in M1 synapses, serving as a crucial cellular mechanism for skill learning and memory retention (Rioult-Pedotti et al., 2000). The indispensable role of dopaminergic projections in M1 becomes evident through experiments where the blockade of dopaminergic projection in M1 leads to a decrease in both long-term potentiation (LTP) and the efficiency of skill learning. Remarkably, despite the attenuation of LTP and skill learning efficiency, synaptic transmission and motor execution remain unaffected (Molina-Luna et al., 2009). In summary, it is deduced that rewarding stimuli activate VTA dopaminergic neurons, thereby regulating downstream components of the reward circuit. Simultaneously, these stimuli transmit a ‘memory’ signal to M1, resulting in the enhancement of memory retention in the context of motor learning (refer to Figure 3).

In totality, the explicit and implicit facets of learning manifest distinct time constants throughout various stages, evident in both sequence and adaptation learning paradigms. The impact of reward on expediting learning speed and augmenting the scope of learning remains inconclusive, exhibiting variability across diverse experimental conditions. Nonetheless, a consensus emerges indicating that reward consistently prolongs both short-term and long-term memory retention in the context of motor learning.

## 5 Limitation and prospects

Although the application of reward in motor repair and rehabilitation shows promise, there exist several limitations that need to be addressed to unlock the full potential of reward on motor learning.

Individual variability in responding to reward stimuli poses a significant challenge. Heterogeneity prevails, as not every individual manifests equivalent sensitivity or responsiveness to rewards, resulting in disparate outcomes in the domain of motor learning. A comprehensive understanding of the factors contributing to this diverseness is imperative, as it forms the bedrock for tailoring personalized reward interventions aligned with an individual’s neural profile. The adoption of customized approaches holds the potential to optimize outcomes in motor rehabilitation. Alternatively, we support the notion that reward systems based on uncertainty outperform those with set amounts in terms of effectiveness, and that rewards given immediately after motor activities are preferable to those awarded later.

The prevalent tendency in current research is to predominantly evaluate short-term effects, resulting in a lack of comprehensive understanding regarding the enduring sustainability of motor improvements and the nuanced impact of rewards at various stages. Subsequent research initiatives ought to prioritize longitudinal studies that meticulously examine the sustained impact of interventions based on rewards. A thorough comprehension of the trajectory of motor improvements over extended periods is imperative for the formulation of interventions that instigate enduring changes in motor function.

The predominant reliance on monetary incentives within experimental designs may inadequately capture the intricacies of real-world motivators. It is essential to explore a more extensive array of rewards, encompassing intrinsic and social incentives, for the development of interventions that resonate with diverse individuals undergoing motor rehabilitation. Virtual reality, robotics, and brain-computer interfaces present promising avenues for augmenting reward-based motor rehabilitation. The integration of rewards with immersive and interactive technologies holds the potential to create interventions that are both engaging and efficacious.

Many investigations center on elementary motor tasks, and the generalizability of their findings to intricate real-world scenarios is constrained. The challenge persists in extrapolating the efficacy of reward-based interventions across a spectrum of motor skills and diverse populations, encompassing individuals with varying degrees of motor impairment. Essential to address is the imperative to bridge the disparity between findings derived from controlled laboratory environments and their practical application in real-world contexts. Subsequent research endeavors should prioritize the development of interventions seamlessly integrated into daily life, ensuring the observed benefits in controlled settings translate into functional enhancements in everyday motor tasks.

Our comprehension of the underlying neurobiological mechanisms linking reward to motor learning remains incomplete. The imperative for rigorous animal experiments, though challenging, is paramount to replicate the effects of reward and elucidate potential neural mechanisms, particularly at the microcircuit level. A pressing need exists for further elucidation of how reward signals are processed within entire neural circuits, particularly in individuals afflicted with neurological disorders. Such insights are foundational for refining targeted interventions and potential modulation of the cortex.

## Author contributions

JZ: Conceptualization, Data curation, Investigation, Writing – original draft, Writing – review & editing. GZ: Visualization, Writing – original draft. DX: Conceptualization, Funding acquisition, Supervision, Validation, Writing – review & editing.
